# Fostering Leadership in Psychiatry: Outcomes of the West Midlands Leadership Programme for Higher Trainees

**DOI:** 10.1192/bjo.2025.10251

**Published:** 2025-06-20

**Authors:** Feroz Nainar, Rosie England, Kieran Groom, Lorna Almond

**Affiliations:** 1 Birmingham Community Healthcare NHS Foundation Trust, Birmingham, United Kingdom; 2 Birmingham and Solihull Mental Health NHS Foundation Trust, Birmingham, United Kingdom

## Abstract

**Aims:** To outline the development and implementation of the West Midlands Psychiatry Leadership Programme, a pioneering initiative believed to be unique within UK psychiatry training, and to evaluate participant feedback on its impact and benefits.

**Methods:** The programme, led by honorary leadership tutors who are higher psychiatry trainees, combines three online training days and a face-to-face conference. Conducted across 2024, it allowed participants time to reflect between sessions. Open to all psychiatry higher trainees in the West Midlands Deanery, invitations were sent via email. Feedback, collected via Microsoft Forms, evaluated its impact in preparing trainees for consultant roles through theory, practical application, self-awareness, and team leadership.

Day 1: Focused on leadership roles within the NHS, exploring operational structures, financial frameworks, emotional intelligence, and personal leadership styles.

Day 2: Addressed “Preparing to be a Consultant” with presentations, scenario analysis, and panel discussions on transitioning to consultancy.

Day 3: Explored “Leading in Teams and Organisations” through organisational change frameworks, team-building exercises, and QI project workshops.

Conference: Provided a platform for trainees to showcase achievements, network, and gain insights from external speakers.

**Results:** Quantitative and qualitative feedback was collected.

Day 1: Overall satisfaction of the day averaged a score of 4.5/5. 48% of attendees rated the content as “very good” and 52% rated it as “good”. Participants praised the interactive breakout rooms and emotional intelligence sessions, with suggestions for enhanced face-to-face elements.

Day 2: Overall satisfaction of the day averaged 4.63/5. More specifically, 77% of attendees were “very satisfied” with the content. The practical consultant preparation talks and scenario-based discussions were highly valued. Feedback emphasised the value of real-world insights.

Day 3: Overall satisfaction of the day averaged 4.52/5. More specifically, 79% of attendees rated the content as “very satisfied”. The engaging role-playing activities and QI workshops were key highlights. Suggestions from feedback included more examples of successful QI projects.

Conference: Overall satisfaction averaged 4.53/5, with 80% reporting that they were “very satisfied” with speakers. 61% stated expectations were met, while 39% said expectations were exceeded. Networking opportunities and personal leadership insights were praised. Suggestions from feedback included a broader range of topics and more external speakers.

**Conclusion:** The programme addresses a critical gap in psychiatry leadership training by blending theory with practice and fostering collaboration. High satisfaction and positive feedback highlight its success. The conference enhances its impact by showcasing achievements and fostering networking. Future iterations will refine content, broaden participation, and assess long-term outcomes.

